# Early unrestricted vs. partial weight bearing after uncemented total hip arthroplasty: a systematic review and meta-analysis

**DOI:** 10.3389/fsurg.2023.1225649

**Published:** 2023-11-15

**Authors:** Li Huang, Weiyu Han, Weizhong Qi, Xiaomeng Zhang, Zhou Lv, Yao Lu, Danfeng Zou

**Affiliations:** ^1^ Department of Joint and Orthopedics, Orthopedic Center, Zhujiang Hospital, Southern Medical University, Guangzhou, China; ^2^ Huiqiao Medical Center, Nanfang Hospital of Southern Medical University, Guangzhou, China

**Keywords:** total hip arthroplasty, uncemented, partial weight bearing, unrestricted weight bearing, rehabilitation

## Abstract

**Background:**

The choice of postoperative weight bearing protocol after uncemented total hip arthroplasty (THA) remains controversial. The aim of this study was to assess the efficacy and safety of immediate unrestricted weight bearing (UWB) compared with partial weight bearing (PWB) in patients undergoing uncemented THA.

**Methods:**

Relevant articles were retrieved from electronic databases. Both randomized controlled trials (RCTs) and non-RCTs were included but analyzed separately. All functional and clinical outcomes with at least 2 independent study outcomes were meta-analyzed.

**Results:**

A total of 17 studies were investigated. No adverse effect was found regarding micromotion of the femoral stem with immediate UWB following uncemented THA. There was also no correlation between immediate UWB and failure of ingrowth fixation and higher risks of femoral stem subsidence and surgical revision in RCTs. Harris hip score was better in patients with immediate UWB than those with PWB at 1 year post surgery, but the difference was not statistically significant.

**Conclusions:**

Immediate UWB did not have extra harm compared with PWB in patients undergoing uncemented THA. UWB was not superior to PWB. Considering the improvement of Harris score and the compliance of patients, UWB can be encouraged in THA rehabilitation.

## 1. Introduction

Total hip arthroplasty (THA) is the most widely performed procedure for end-stage joint diseases (1). Patient satisfaction-related outcomes, regarding pain relief, functional recovery, and improvement in mobility and quality of life, have been reported by both patients and physicians after THA (2). Cemented and uncemented prostheses are two choices of fixation for THA. Although cemented THA could provide relatively better prognosis for elderly patients (3), increasing early loosening rates have been reported (4, 5). Besides, younger patients who underwent cemented THA have exhibited higher risk of revision due to more exercise (6). Moreover, uncemented or hybrid fixation can improve survivorship in younger patients while cemented fixation are better for older patients (7–9). A recent single-center survival analysis involved 2,156 hips also reveals that uncemented THA show improved survival over cemented at younger ages (10). Thus, the use of uncemented THA has been increasingly recommended in recent years. With the increased in life expectancy along with the change of thresholds for surgery, the number of uncemented THAs is expected to raise more rapidly.

Partial weight bearing (PWB) for 6–12 weeks is still advocated for patients undergoing uncemented THA to create optimal requirements for bone and soft tissue healing as well as to reduce implant failures (11–13). However, this recommendation is frequently based on empirical belief instead of on evidence from the literature. Modern postoperative management is becoming more focused on techniques that facilitate early physiological rehabilitation, including early weight-bearing activities, functional exercises, and muscle exercises. Some studies propose that instead of negative influence on implant stability or clinical results, postoperative immediate unrestricted weight bearing (UWB) could shorten hospital stay, accelerate functional recovery, improve muscle strength, provide higher autonomy, and prevent complications (e.g., deep leg vein thrombosis, urinary tract infections, and pneumonia) (14–17). As to rehabilitation of THA, early postoperative exercise under the premise of safety is well-recognized. However, there is no uniform standard for the degree of weigh bearing. Moreover, it's still unclear that how postoperative weight bearing affect the outcome of THA.

At present, no clear evidence exists on the most optimal physical rehabilitation protocols after uncemented THA, and controversies exist on whether to use UWB or PWB procedures. Thus, the scope of the present meta-analysis was to compare the efficacy and safety of UWB and PWB in patients undergoing uncemented THA, thereby identifying the evidence-based guidelines that can be used in clinical practice.

## 2. Methods

### 2.1. Search strategies

The current meta-analysis was conducted according to the Preferred Reporting Items for Systematic reviews and Meta-Analyses (PRISMA) statement (18). Comprehensive searches of PubMed, EMBASE, Web of Science, and Cochrane Library were undertaken using Mesh headings and text words for hip arthroplasty and weight bearing. The search terms were kept broad to cover all the possibilities. To expand the search for additional articles of interest, the bibliography of all studies included in this analysis were manually cross-checked. There was no restriction on the publication date or language. All potentially eligible publications were evaluated for inclusion independently by two reviewers based on the title, abstract, and full-text articles when necessary. Conflicts on eligibility were resolved by discussion.

### 2.2. Selection criteria

Studies were eligible for inclusion if they met the following criteria: (1) comparative studies including randomized controlled trials (RCTs) or cohort studies; (2) all patients who underwent primary uncemented THA, or data from the subgroup with uncemented THA were analyzed separately; (3) comparing the effect between UWB and PWB after uncemented THA. Studies assessing patients with cemented THA or revision of the THA, abstracts, reviews, and case reports, were excluded. In case of duplicate publications with overlapping patient data, only the most recent or informative one was included.

### 2.3. Data extraction

Relevant information and outcome data were extracted by two reviewers independently according to a predefined standardized form. The items extracted from the included studies were as follows: study originations (first author, publication year, region of experiment), participants (number, age, gender, clinical characteristics, surgical approach, and prosthetic design), interventions (level of weight bearing, use of assistive devices, duration, and follow-up time), and outcomes. All data were checked for missing value, consistency, and validity.

### 2.4. Quality assessment

Quality assessment was performed using the Physiotherapy Evidence Database (PEDro) scale for RCTs (19) and the index for non-randomized studies (MINORS) form for non-RCTs (20). The PEDro scale is a reliable tool developed to rate the quality of RCTs evaluating physical therapist interventions. It consists of a checklist of 11 criteria, 10 of which are scored. For this analysis, studies with PEDro scores of 6–10 were considered high quality, of 4–5 were considered moderate quality, and of 0 to 3 were considered low quality. The MINORS scale contains 12 items, and the items are scored 0 (not reported), 1 (reported but inadequate) or 2 (reported and adequate). For this analysis, studies with MINORS scores of 19–24 were considered high quality, of 13–18 were considered moderate quality, and of 0–12 were considered low quality.

### 2.5. Statistical analysis

Stata software version 15.0 (Stata Corporation, College Station, TX, USA) was used to perform the statistical analyses. Continuous outcomes were pooled as weighted mean difference (WMD) or standard mean difference (SMD) with 95% confidence interval (CI). Dichotomous outcomes were expressed as odds ratio (OR) with 95% CI. Heterogeneity among the included studies was calculated by Chi-squared *Q* test and *I*^2^ statistics. A random-effects model was chosen significant heterogeneity was identified (*P* value of *Q* test <0.05 or *I*^2^ > 50%). A fixed-effects model was employed if there was no evidence of heterogeneity (*P* > 0.05 and *I*^2 ^< 50%). Sensitivity analysis was conducted by removing each study one by one to test if a particular study altered the overall effect or disproportionately contributed to the observed heterogeneity. Both RCTs and non-RCTs were included in the present meta-analysis, outcomes from different study design were pooled separately. Subgroup analysis based on follow-up time was performed when each subgroup contained at least 2 independent study outcomes. Funnel plots were used for testing publication bias when the number of the included studies exceeded ten. A *P*-value <0.05 was considered statistically significant.

## 3. Results

### 3.1. Search results

**Figure 1:**
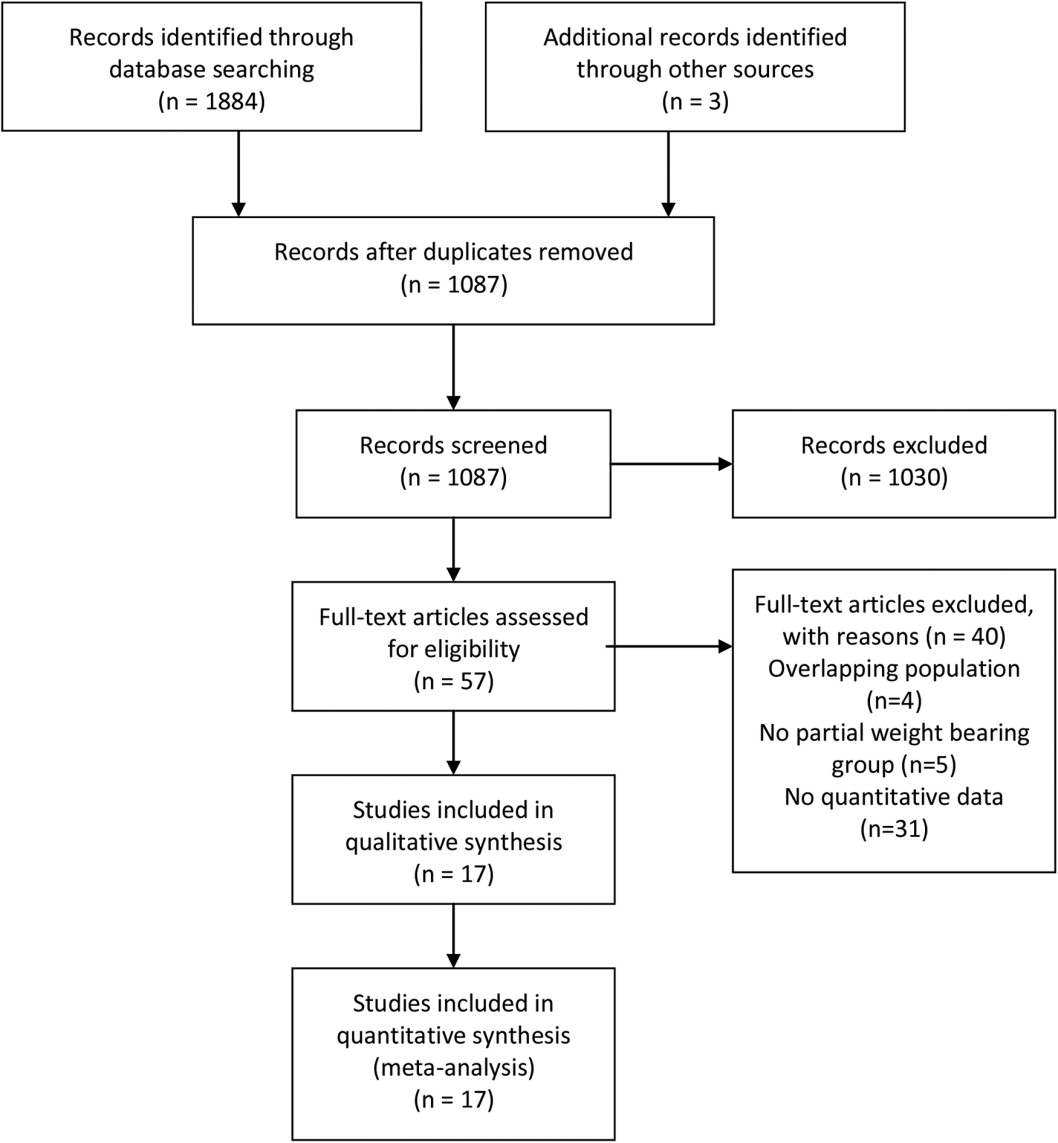
PRISMA flow diagram of literature search.

Overall, electronic database searches led to 1,087 articles after removal of duplicates. Three additional publications were found by reference review. Fifty-seven studies were selected for full-text review. Seventeen studies fulfilling all inclusion criteria and with sufficient outcome data were finally included in the meta-analysis. The process of literature search and study selection were described in Figure 1.

### 3.2. Study characteristics

Ultimately, thirteen RCTs and four retrospective studies were eligible for inclusion (15, 16, 21–29). Most of the studies had small sample size ranged from 20 to 100 hips, only one study included more than 100 patients (21). Five studies included patients undergoing unilateral THA, four studies involved both unilateral and bilateral, while the other 10 studies did not give specific information. Surgical approach and hip type were diverse among studies. Although the specific protocols were different, essentially, patients in UWB group were instructed to bear full weight immediately after surgery without external support or with the use of cane only for balance. Patients in PWB group were allowed to bear 20–50 lb of weight for 6 weeks to 3 months using crutches. Detailed weight bearing protocols and study information were presented in Table 1. According to the quality assessment results, all the included studies obtained moderate to high quality. Distributions of quality assessment in each study were presented in Supplementary Table 1.

**Table 1:** Characteristics of included trials comparing UWB to PWB for postoperative rehabilitation of THA.

Author	Year	Region	Study design	Hip type	Surgical approach	Prosthesis design	Group	*N*	Age	Male/female	Weight bearing protocol
Bernasek	(21)	USA	Retro	−	Direct lateral (modified Hardinge) approach	Proximally porous-coated, tapered, collarless titanium stem (Pinnacle System; DePuy, Warsaw, Indiana)	UWB	146	64 (29–86)	77/69	Walk initially with weight bearing to tolerance with 2-arm support and to progress to a cane for balance as soon as they felt comfortable.
							PWB	163	63 (33–87)	67/96	Allowed 20 lb weight bearing using 2-arm support (a walker at all times) for 6 weeks. Active abduction and ﬂexion
Boden	(22)	Sweden	RCT	Unilateral	−	Hydroxyapatite-coated, tapered stem (Bi-Metric femoral stem and Romanus cup, Biomet Inc., Warsaw, Indiana, USA)	UWB	10	54 (44–59)	−	Carry full weight on the operated leg for 6 s with support only of their balance. They were told to walk with 1 crutch alone or without external support, when possible.
							PWB	10	55 (44–63)	−	Allowed 10% of the body weight by using 2 crutches for 3 months
Bottner	(23)	Sweden	RCT	Unilateral and bilateral	Posterior approach	Porous-coated titanium elliptical cup with a preassembled liner and a Proxilock hydroxyapatite-coated hip stem	UWB	12	46 (35–59)	11/1	−
							PWB	17	47 (24–59)	13/4	−
Chan	(24)	China	Retro	−	−	Hydroxyapatite-coated gritblasted, collarless, straight titanium-alloy femoral implant (Omnifit HA, Osteonics, Stryker, USA)	UWB	29	49.5 ± 16.3	17/12	Allowed to walk immediately with full weight bearing.
							PWB	29	50.5 ± 14.4	17/12	Allowed to walk for 6 weeks with protected weight bearing after surgery.
Kishida	(15)	Japan	RCT	−	−	Spongy metal Lübeck hip prosthesis	UWB	17	52.0 ± 13.0	6/11	Bear full weight on the second day after operation.
							PWB	16	51.0 ± 12.0	4/12	Instructed to maintain touchdown weight-bearing until 3 weeks after surgery, then increase partial weight-bearing over the next 3 weeks.
Markmiller	(25)	Germany	RCT	-	Anterior or transgluteal approach	Hydroxyapatite-coated Spotorno-type femoral shaft component and a cementless titanium-coated acetabular component	UWB	50	60.6 ± 12.5	19/31	Immediately instructed to walk without external support whenever possible.
							PWB	50	61.2 ± 13.1	22/28	Allowed to walk with 15 kg weight on the operated hip using crutches for 6 weeks.
Matheis	(26)	Germany	RCT	−	Modified anterolateral approach	−	UWB	20	65.5 ± 7.4	13/7	−
							PWB	19	66.7 ± 9.8	22/28	−
Monticone	(27)	Italy	RCT	−	−	−	UWB	47	69.5 ± 7.5	18/32	Instructed to use their crutches reciprocally to regain a symmetrical gait pattern, but were also encouraged to abandon any walking aids by the end of their in-hospital stay.
							PWB	48	68.8 ± 8.1	22/28	Instructed to use their crutches reciprocally, allowed to use partial weight-bearing on the operated limb, and recommended to use walking aids for three months after surgery.
Rao	(16)	USA	Retro	Unilateral and bilateral	Modified Hardinge or transtrochanteric approach	Uncemented Taperloc (Biomet, Warsaw, IN) femoral prosthesis and an uncemented acetabular cup (Modified Universal, Biomet, Warsaw, IN).	UWB	14	52 (37.8–67.4)	6/8	Allowed to bear weight on both lower limbs as tolerated with the help of two crutches or a walker. Use of the walker or crutches was continued for 6 weeks after surgery, at which time a cane was advised.
							PWB	28	55 (26.3–80.2)	12/16	Allowed 10% weightbearing on the surgically treated limb for 6 weeks after surgery, at which time these patients were allowed to bear weight as tolerated with a cane.
Shabana	(28)	Egypt	RCT	Unilateral and bilateral	−	−	UWB	10	54.5 (50–65)	5/5	Allowed to use a cane or one crutch in the first week or within the hospital stay only for balance not for weight bearing.
							PWB	10	56 (51–65)	5/5	Started with graduated weight bearing (GWB) gait training.
Strom	(29)	Sweden	RCT	unilateral	Anteriolateral approach	Uncemented CLS hip stem (Centerpulse™, Bern, Switzerland).	UWB	16	54.2	9/7	Encouraged to participate in unrestricted early weightbearing from the first postoperative day combined with intensive physiotherapy training during the first 3 months. The patients in the UWB group were allowed to use crutches if needed.
							PWB	13	55.3	6/7	Instructed to walk with a load of approximately 15 kg on the surgically treated leg (to walk with a load corresponding to the weight of the leg) for 3 months
Strom	(30)	Sweden	RCT	Unilateral	Anteriolateral approach following Hardinge	uncemented CLS hip stem (Centerpulse™, Bern, Switzerland).	UWB	21	54.5	12/9	Encouraged to participate in unrestricted early weightbearing from the first postoperative day combined with intensive physiotherapy training during the first 3 months. The patients in the UWB group were allowed to use crutches if needed.
							PWB	21	55.6	10/11	Instructed to walk with a load of approximately 15 kg on the surgically treated leg (to walk with a load corresponding to the weight of the leg) for 3 months
Thien	(31)	Sweden	RCT	−	−	Uncemented and hydroxyapatite-coated prosthesis with an anteverted stem (ABG I; Stryker-Howmedica)	UWB	21	53 (46–60)	11/10	Immediately instructed to walk with 1 crutch alone or without external support whenever possible.
							PWB	19	54 (41–63)	10/9	Allow protected weight bearing using 2 crutches and using the auditory device for feedback.
Unver	(32)	Turkey	RCT	Unilateral and bilateral	Lateral approach	Thrust plate prosthesis	UWB	24	49.9 ± 10.0	6/16	Accelerated rehabilitation with full weight bearing the day after surgery and repeated twice a day.
							PWB	27	48.9 ± 12.9	7/20	Accelerated rehabilitation with partial weight bearing.
Wolf	(33)	Sweden	RCT	Unilateral	Anterolateral approach	CLS hip stem (Centerpulse, now Zimmer Co, Warsaw, IN, USA) with a 28-mm cobalt–chrome head	UWB	18	59 ± 2.6	10/8	Instructed to bear full weight directly after surgery for 3 months after surgery.
							PWB	20	53 ± 9.6	10/10	Instructed to bear weight partially, approximately 15 kg, for 3 months.
Wolf	(34)	Sweden	RCT	Unilateral	Anterolateral approach	CLS hip stem (Centerpulse, now Zimmer Co, Warsaw, IN, USA) with a 28-mm cobalt–chrome head	UWB	13	53 ± 12	6/7	Instructed to bear full weight directly after surgery for 3 months after surgery.
							PWB	17	54 ± 8	8/9	Instructed to bear weight partially, approximately 15 kg, for 3 months.
Woolson	(35)	USA	Retro	−	Posterolateral approach	Extensively porous-coated femoral component without cement [Anatomic Medullary Locking (AML) or Solution femoral prostheses; DePuy, Warsaw, IN].	UWB	24	65 (44–73)	14/11	Instructed to be full weight bearing immediately after the operation using 2 crutches for balance only and were allowed to switch to 1 crutch or cane whenever they felt comfortable with 1 support aid.
							PWB	24	54 (33–75)	19/6	Instructed to bear 50 lb of weight on their operated hip for 6 weeks using 2 crutches followed by progression to full weight bearing over the subsequent 4 weeks.

Retro, retrospective cohort; RCT, randomized controlled trial; Age of patients is presented as mean (minimum–maximum) or mean ± standard deviation.

### 3.3. Stem micromotion

Migration of stem was measured in five studies using radiostereometric analysis (RSA). Significant difference between the UWB and PWB groups occurred at 1-week, 1-month, and 3-month, as a difference in the medial (+) or lateral (–) migration of the stem, as well as at 1-month and 3-month follow-up in anterior (+) or posterior (–) migration of the stem (Table 2). There was no significant difference in proximal (+) or distal (–) subsidence of the stem between groups; nor was there any significant difference in anterior or posterior tilt, retroversion or anteversion, and valgus or varus tilt between groups (Table 2).

**Table 2:** Summary of stem micromotion measured by radiostereometric analysis.

Group	*N*	WMD	95% CI	*P*	*I* ^2^
X-translation, mm, medial+/lateral−					
1-week	2	−0.07	−0.11, −0.03	0.001	0%
1-month	4	−0.06	−0.10, −0.02	0.014	0%
3-month	4	−0.09	−0.16, −0.02	0.016	0%
1-year	5	−0.03	−0.08, 0.03	0.314	37.8%
2-year	3	0.03	−0.08, 0.13	0.625	0%
5-year	2	−0.20	−0.45, 0.04	0.106	0%
Y-translation, mm, proximal+/distal−					
1-week	2	−0.03	−0.08, 0.02	0.253	0%
1-month	5	0.05	−0.17, 0.26	0.677	0%
3-month	6	0.13	−0.16, 0.41	0.375	0%
1-year	5	−0.24	−0.68, 0.21	0.298	0%
2-year	3	−0.67	−2.36, 1.03	0.441	89.8%
5-year	2	−1.42	−5.20, 2.37	0.464	93.6%
Z-translation, mm, anterior+/posterior−					
1-week	2	0.02	−0.04, 0.08	0.472	0%
1-month	4	0.13	0.06, 0.20	< 0.001	0%
3-month	4	0.21	0.12, 0.30	< 0.001	22.2%
1-year	5	0.09	−0.02, 0.20	0.104	0%
2-year	3	0.01	−0.17, 0.18	0.973	63.3%
5-year	2	−0.07	−0.43, 0.30	0.727	0%
X-rotation (°), anterior+/posterior− tilt					
1-week	2	0.01	−0.08, 0.08	0.961	13.5%
1-month	4	−0.08	−0.19, 0.03	0.134	0%
3-month	5	0.03	−0.09, 0.15	0.598	0%
1-year	5	0.03	−0.15, 0.22	0.720	0%
2-year	3	−0.18	−0.42, 0.07	0.158	0%
5-year	2	−0.20	−0.78, 0.37	0.485	33.5%
Y-rotation (°), retroversion+/anteversion−					
1-week	2	0.03	−0.12, 0.18	0.696	81.7%
1-month	4	−0.10	−0.44, 0.24	0.572	0%
3-month	5	−0.24	−0.71, 0.23	0.318	0%
1-year	5	0.45	−0.18, 1.08	0.160	0%
2-year	3	0.52	−0.18, 1.21	0.147	0%
5-year	2	0.07	−1.17, 1.32	0.907	35.5%
Z-rotation (°), valgus+/varus− tilt					
1-week	2	−0.04	−0.10, 0.03	0.263	0%
1-month	3	−0.05	−0.15, 0.05	0.352	33.7%
3-month	3	−0.06	−0.24, 0.12	0.495	71.3%
1-year	3	−0.12	−0.33, 0.10	0.281	0%
2-year	2	−0.10	−0.34, 0.13	0.381	0%

WMD, weighted mean difference.

### 3.4. Stem stability

Bone ingrowth fixation was evaluated in four RCTs and three non-RCTs based on the Engh criteria (36). Overall, the incidence of bone ingrowth fixation did not differ significantly between the UWB and PWB groups. As to RCTs, Markmiller et al. and Bottner et al. find that all cases achieve bone ingrowth fixation (23, 25). Bodén et al. shows that the bone ingrowth fixation rate is 90% in both UWB and PWB groups (22). In non-RCTs, bone ingrowth occurs in all three researches.

**Figure 2:**
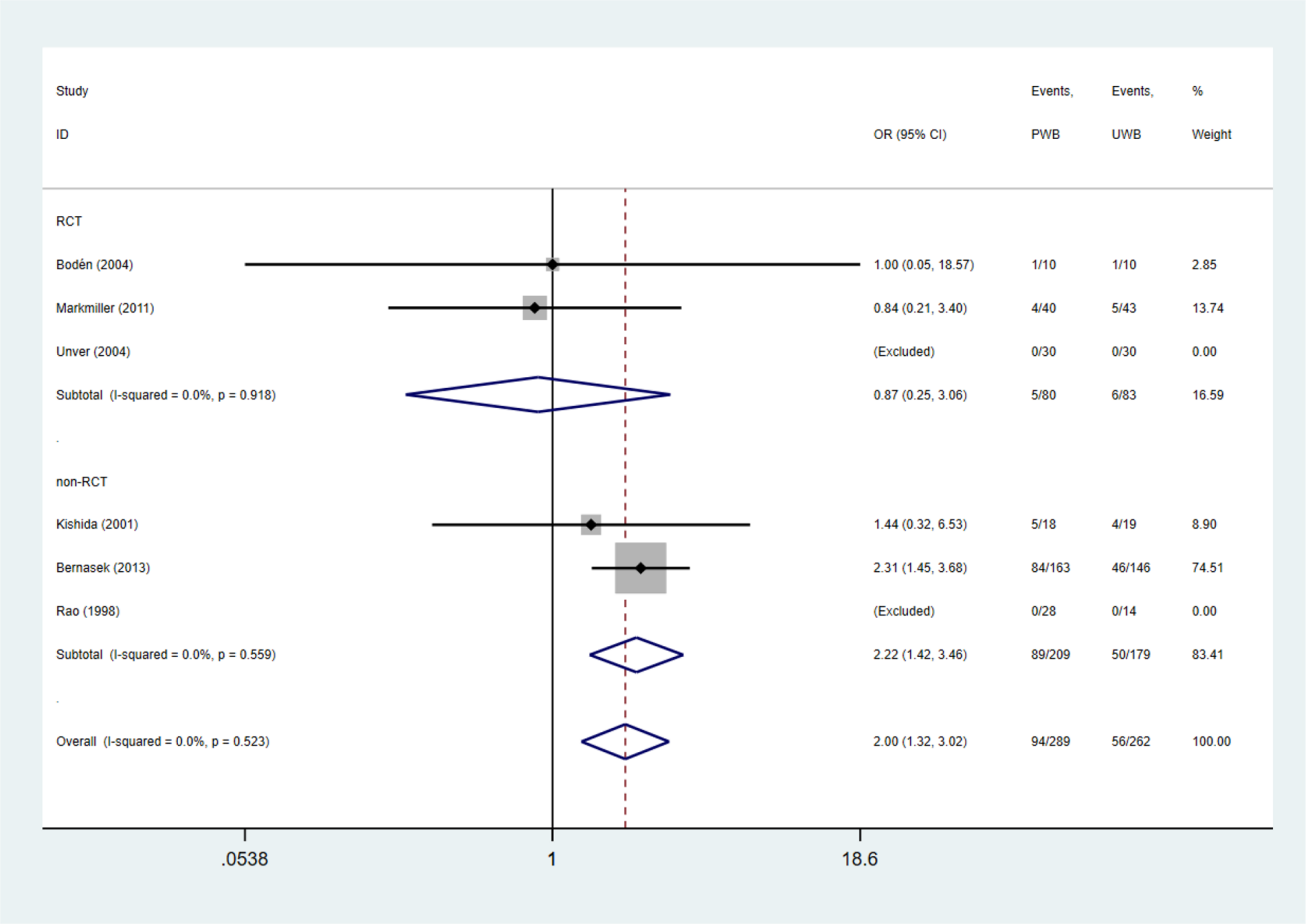
Forest plots of radiolucent lines.

Radiolucent lines were assessed in three RCTs and non-RCTs, respectively. The incidence of radiolucent lines was higher in UWB group than that in PWB group in non-RCTs (OR = 2.22, 95% CI, 1.42, 3.46; *P* = 0.00; *I*^2^ = 0%). However, in RCTs with more rigorous design, no significant difference was found between groups (OR = 0.87, 95% CI, 0.25, 3.06; *P* = 0.830; *I*^2^ = 0%; Table 3 and Figure 2).

**Table 3:** Mean differences (95% CI) for stem stability in trials comparing UWB to PWB.

Group	*N*	OR	95% CI	*P*	*I* ^2^
Radiolucent lines					
RCT	3	0.87	0.25, 3.06	0.830	0%
Non-RCT	3	2.22	1.42, 3.46	0.000	0%
Femoral component subsidence (>1 mm)					
RCT	4	1.55	0.46, 5.21	0.477	0%
Non-RCT	3	0.58	0.36, 0.93	0.023	53.6%

OR, odds ratio.

**Figure 3:**
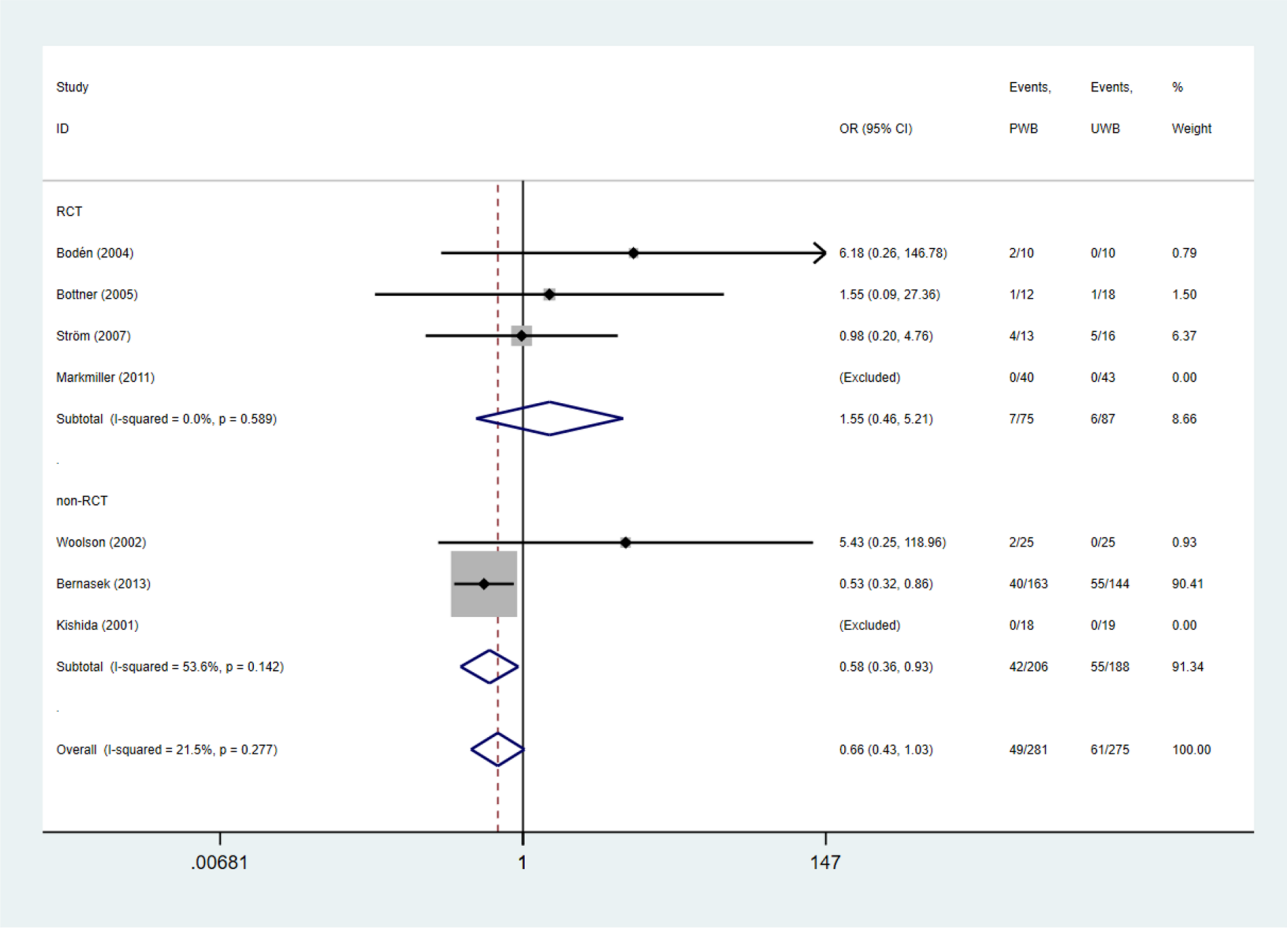
Forest plots of femoral component subsidence.

Femoral component subsidence (>1 mm) was evaluated in four RCTs and three non-RCTs. The incidence of femoral component subsidence (>1 mm) did not differ significantly between the UWB and PWB groups (RCTs: OR = 1.55, 95% CI, 0.46, 5.21; *P* = 0.477; *I*^2^ = 0%). Whereas, UWB group exhibited a lower incidence of femoral component subsidence relative to PWB group in non-RCTs (OR = 0.58, 95% CI, 0.36, 0.93; *P* = 0.023; *I*^2^ = 53.6%; Table 3 and Figure 3).

### 3.5. Complications

**Figure 4:**
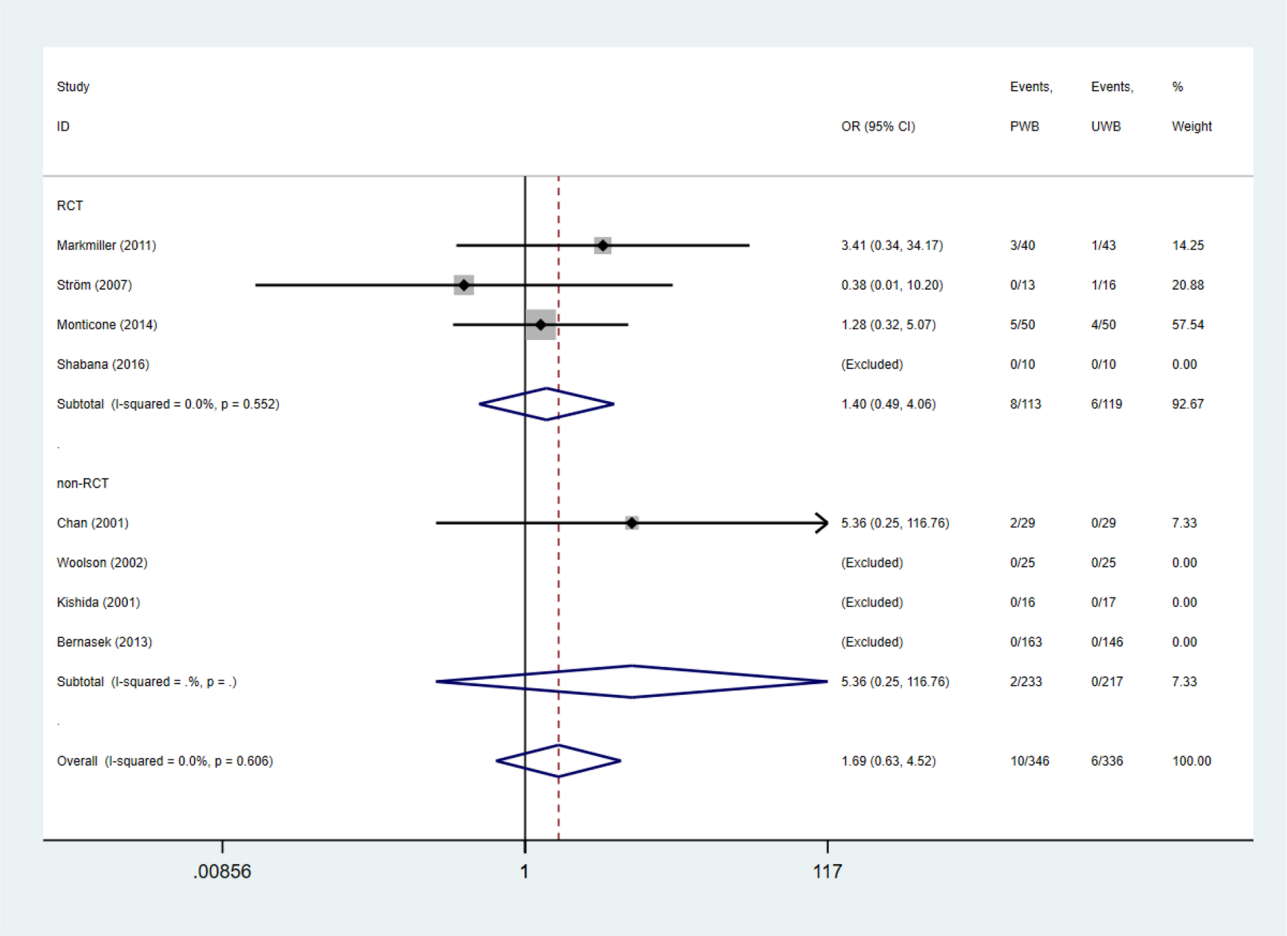
Forest plots of complications.

The incidences of symptomatic deep venous thrombosis, infection, dislocation, and surgical revision were also estimated (37). No significant difference was found between the UWB and PWB groups regarding the abovementioned complication (Table 4 and Figure 4).

**Table 4:** Mean differences (95% CI) for postoperative complications in trials accessing the safety of UWB versus PWB.

Group	*N*	OR	95% CI	*P*	*I* ^2^
RCT	4	1.40	0.49,4.06	0.532	0%
Non-RCT	4	5.36	0.25, 116.76	0.285	0%
Total	8	1.69	0.63, 4.52	0.606	0%

OR, odds ratio.

### 3.6. Harris Hip score

Four RCTs and two retrospective studies provided enough data to calculate the difference between pre- and postoperative Harris hip score. Pooled results showed that UWB group improved Harris hip score by 2.27 (95% CI, −0.96, 5.49; *P* = 0.169; *I*^2^ = 0%) in RCTs and by 2.63 (95% CI, −2.59, 7.84; *P* = 0.323; *I*^2^ = 20.2%) in non-RCTs compared with PWB group (Table 5). However, the improvement did not reach statistical significance.

**Table 5:** Results of harris Hip score.

Group	*N*	WMD	95% CI	*P*	*I* ^2^
RCT	4	2.27	−0.96, 5.49	0.169	0%
3-month	2	5.37	−8.30, 19.03	0.442	85.2%
>1-year	3	3.34	−0.33, 7.00	0.074	0%
Non-RCT	2	2.63	−2.59, 7.84	0.323	20.2%

WMD, weighted mean difference.

Subgroup analysis was conducted with data from RCTs. No significant difference was found between UWB and PWB groups in Harris hip score at 3-month and ≥1-year follow-up (Table 5).

### 3.7. Sensitivity analysis

Sensitivity analysis was performed in all assessed outcomes. In all parameters, no individual study was found to alter the direction and size of the overall effect size.

## 4. Discussion

The optimum form of postoperative weight bearing protocol after uncemented THA remains controversial. It is concerned that PWB following uncemented THA may inhibit functional recovery and increase the risk of complications (9, 38), whereas UWB may cause micromovement at the bone-implant interface, jeopardizing stability, and ingrowth of implant (39). By integrating data from 17 studies, this meta-analysis established some levels of evidence to support the use of immediate UWB after uncemented THA. There was no statistically significant difference between UWB and PWB groups regarding micromotion of the femoral stem, ingrowth fixation, femoral component subsidence, revision, and complications. In RCTs, the outcome of Harris hip score in the UWB group was better than that in the PWB group at 1 year post surgery, but the difference was not statistically significant (*P* = 0.074).

A previous meta-analysis shows greater proximal or distal femoral stem subsidence in UWB compared with PWB groups at 3-month follow-up (40), which is contradict with our findings. In the analysis of femoral stem micromotion, the present study only included RCTs, and analyzed data of the CLS femoral stem along and around the three axes measured with RSA. Whereas Tian et al. integrated both RCTs and non-RCTs, and included data measured with conventional radiographs (41). Thus, our study may be more validity. In addition, significant differences were found in medial or lateral translation and anterior or posterior translation at 1- and 3-month follow-up, while these significant differences did not maintain at further measurement. The initial stability of the uncemented implants is dependent on the mechanical match between the prosthetic stem and the intramedullary canal. The long-term stability is determined by mechanical fit and bone ingrowth (25). If the initial mechanical match has not been achieved, the femoral stem prosthesis will descend along the medullary cavity in the weight bearing activities until a tight matching occur (16). Thus, in the first postoperative three months, the higher femoral subsidences of patients in the UWB group might be because the prosthesis and the medullary cavity did not achieve the best matching. When the weight bearing increased gradually in the PWB group, the femoral stem subsidence began to catch up, and thus the femoral subsidences of the two groups tended to be consistent at one or more years after THA.

Femoral stem subsidence greater than 1–1.5 mm during the first two years after uncemented THA has been shown to predict an increased risk of early or midterm revision (29). In this meta-analysis, we showed that there was no significant difference of the test results of UWB compared with PWB groups in the incidences of femoral component subsidence (>1 mm), and subsequently the incidences of revision in four RCTs. These findings added support for allowing UWB after uncemented THA.

Several included studies use an auditory device calibrated to between 10% of body weight to 30 kg of loading to instruct the patients (22, 29–31, 33, 34). However, some patients do not strictly follow the instruction to full extent. In some studies, patients in the PWB group put almost twice the recommended weight on the operated leg (29, 33, 34). This might contribute to the insignificant test results between UWB and PWB groups. Thus, further studies with more rigorous design are needed to verify our findings.

Several potential limitations should be noted. Publication bias could not be tested by Deeks funnel plot and Egger's asymmetry testing due to extremely limited number of studies in each outcome. Although 17 publications were included, some of the analyses only involved a small number of studies with small sample sizes, which might be insufficient to draw definite conclusion. The influence of some confounding factors, which have been suggested to be important factors in stability and ingrowth (i.e., such as prosthesis design (16, 42, 43), surgical approach (21, 36), and the use and duration of assistive devices (12, 32)), could not be controlled due to limited amount of data. Thus, results from the present meta-analysis should be interpreted with caution. Future prospective, multi-institutional, well-designed trials with larger sample size are needed to testify our results.

Several reasons may be responsible for the limited investigations on the effects of immediate UWB after uncemented THA. There is inherent fear that allowing patients to bear weight unrestrictedly may lead to higher risk of subsidence and revision. Thus, it may seem unethical to randomize patients between groups with different weight bearing regimes if the risk of one group is a failure of fixation. The current meta-analysis revealed no statistically significant evidence of additional harm of immediate UWB after uncemented THA. This finding may eliminate some of the doubt of immediate UWB after uncemented THA, and provide evidence-based support to encourage more future studies on this topic.

## 5. Conclusion

In conclusion, within the current literature, immediate UWB did not have extra harm and might have potential benefit in functional outcomes compared with PWB in patients who underwent uncemented THA. UWB was not superior to PWB. Considering the improvement of Harris score and the compliance of patients, UWB can be encouraged in THA rehabilitation.

## Data Availability

The original contributions presented in the study are included in the article/**Supplementary Material**, further inquiries can be directed to the corresponding authors.
