# Supplementary material for: Early unrestricted vs. partial weight bearing after uncemented total hip arthroplasty: a systematic review and meta-analysis
Source: Front Surg. 2023 Nov 15;10:1225649. doi: 10.3389/fsurg.2023.1225649 (PMC10684916; doi:10.3389/fsurg.2023.1225649)
Supplement: Supplementary file 1 [file Table1.docx]

Supporting Information

**Early unrestricted versus partial weight bearing after uncemented total hip arthroplasty: a systematic review and meta-analysis**

Li Huang^1^, Weiyu Han^1^, Weizhong Qi^1^, Xiaomeng Zhang^1^, Zhou Lv^1^, Yao Lu^1,^*, Danfeng Zou^2,^*

1. Department of Joint and Orthopedics, Orthopedic Center, Zhujiang Hospital, Southern Medical University, Guangzhou, Guangdong 510282, China.

2. Huiqiao Medical Center, Nanfang Hospital of Southern Medical University, Guangzhou, Guangdong 510515, China.

*Corresponding Authors: Yao Lu (E-mail: oayul@smu.edu.cn); Danfeng Zou (E-mail: 13609060636@139.com)

**Supplementary Table 1: Quality assessment results.**

PEDro-Physiotherapy Evidence Database scale

| **Author** | **Year** | **1** | **2** | **3** | **4** | **5** | **6** | **7** | **8** | **9** | **10** | **11** | **Total** | **Quality** |
| --- | --- | --- | --- | --- | --- | --- | --- | --- | --- | --- | --- | --- | --- | --- |
| Boden | 2004 | 1 | 1 | 1 | 1 | 0 | 0 | 1 | 1 | 1 | 1 | 1 | 8 | High |
| Bottner | 2005 | 1 | 0 | 0 | 1 | 0 | 0 | 1 | 1 | 1 | 1 | 1 | 6 | High |
| Kishida | 2011 | 0 | 1 | 0 | 1 | 0 | 0 | 0 | 1 | 1 | 1 | 1 | 6 | High |
| Markmille | 2010 | 0 | 1 | 1 | 1 | 0 | 0 | 1 | 1 | 1 | 1 | 1 | 8 | High |
| Matheis | 2017 | 1 | 1 | 0 | 1 | 0 | 0 | 0 | 1 | 1 | 1 | 1 | 6 | High |
| Monticone | 2014 | 1 | 1 | 1 | 1 | 1 | 0 | 1 | 0 | 1 | 1 | 1 | 8 | High |
| Shabana | 2017 | 1 | 0 | 0 | 1 | 0 | 0 | 0 | 0 | 1 | 1 | 1 | 4 | Moderate |
| Strom | 2006 | 1 | 1 | 1 | 1 | 0 | 0 | 1 | 1 | 1 | 1 | 1 | 8 | High |
| Strom | 2007 | 1 | 1 | 1 | 1 | 0 | 0 | 0 | 1 | 1 | 1 | 1 | 7 | High |
| Thien | 2007 | 1 | 1 | 1 | 1 | 0 | 0 | 0 | 1 | 1 | 1 | 1 | 7 | High |
| Unver | 2004 | 1 | 1 | 0 | 1 | 0 | 0 | 1 | 1 | 1 | 1 | 1 | 7 | High |
| Wolf | 2010 | 1 | 1 | 0 | 0 | 0 | 0 | 0 | 1 | 1 | 1 | 1 | 5 | Moderate |
| Wolf | 2012 | 1 | 1 | 0 | 1 | 0 | 0 | 0 | 0 | 1 | 1 | 1 | 5 | Moderate |

**“1” for “yes”; “0” for “no”.
Ranking criteria: 6-10 scores for “High”; 4-5 scores for “Moderate”; 0-3 scores for “Low”.**

**Item 1-**Eligibility criteria were specified;
**Item 2-**Subjects were randomly allocated to groups (in a crossover study, subjects were randomly allocated an order in which treatments were received);
**Item 3-**Allocation was concealed;
**Item 4-**The groups were similar at baseline regarding the most important prognostic indicators;
**Item 5-**There was blinding of all subjects;
**Item 6-**There was blinding of all therapists who administered the therapy;
**Item 7-**There was blinding of all assessors who measured at least one key outcome;
**Item 8-**Measures of at least one key outcome were obtained from more than 85% of the subjects initially allocated to groups;
**Item 9-**All subjects for whom outcome measures were available received the treatment or control condition as allocated or, where this was not the case, data for at least one key outcome was analyzed by “intention to treat”;
**Item 10-**The results of between-group statistical comparisons are reported for at least one key outcome;
**Item 11-**The study provides both point measures and measures of variability for at least one key outcome.

MINORS- the index for non-randomized studies form

| **Author** | **Year** | **1** | **2** | **3** | **4** | **5** | **6** | **7** | **8** | **9** | **10** | **11** | **12** | **Total** | **Quality** |
| --- | --- | --- | --- | --- | --- | --- | --- | --- | --- | --- | --- | --- | --- | --- | --- |
| Bernasek | 2013 | 2 | 2 | 0 | 1 | 0 | 2 | 2 | 2 | 2 | 0 | 0 | 2 | 15 | Moderate |
| Chan | 2003 | 2 | 2 | 0 | 1 | 0 | 2 | 2 | 2 | 2 | 0 | 2 | 2 | 17 | Moderate |
| Rao | 1998 | 2 | 2 | 1 | 2 | 0 | 2 | 2 | 2 | 2 | 1 | 2 | 2 | 20 | High |
| Woolson | 2002 | 2 | 2 | 1 | 2 | 1 | 2 | 2 | 2 | 2 | 1 | 2 | 2 | 21 | High |

**“2” for reported and adequate; “1” for reported but inadequate; “0” for not mentioned or can’t be confirmed.
Ranking criteria: 19-24 scores for “High”; 13-18 scores for “Moderate”; 0-12 scores for “Low”.**

**Item 1-**A clearly stated aim: the question addressed should be precise and relevant in the light of available literature;
**Item 2-**Inclusion of consecutive patients: all patients potentially fit for inclusion (satisfying the criteria for inclusion) have been included in the study during the study period (no exclusion or details about the reasons for exclusion);
**Item 3-**Prospective collection of data: data were collected according to a protocol established before the beginning of the study;
**Item 4-**Endpoints appropriate to the aim of the study: unambiguous explanation of the criteria used to evaluate the main outcome which should be in accordance with the question addressed by the study. Also, the endpoints should be assessed on an intention-to-treat basis;
**Item 5-**Unbiased assessment of the study endpoint: blind evaluation of objective endpoints and double-blind evaluation of subjective endpoints. Otherwise, the reasons for not blinding should be stated;
**Item 6-**Follow-up period appropriate to the aim of the study: the follow-up should be sufficiently long to allow the assessment of the main endpoint and possible adverse events;
**Item 7-**Loss to follow up less than 5%: all patients should be included in the follow up. Otherwise, the proportion lost to follow up should not exceed the proportion experiencing the major endpoint;
**Item 8-**Prospective calculation of the study size: information of the size of detectable difference of interest with a calculation of 95% confidence interval, according to the expected incidence of the outcome event, and information about the level for statistical significance and estimates of power when comparing the outcomes;
**Item 9-**An adequate control group: having a gold standard diagnostic test or therapeutic intervention recognized as the optimal intervention according to the available published data;
**Item 10-**Contemporary groups: control and studied group should be managed during the same time period (no historical comparison);
**Item 11-**Baseline equivalence of groups: the groups should be similar regarding the criteria other than the studied endpoints. Absence of confounding factors that could bias the interpretation of the results;
**Item 12**-Adequate statistical analyses: whether the statistics were in accordance with the type of study with calculation of confidence intervals or relative risk**.**

**Stata commands for Table 3 and Figure 2.**

*(10 variables, 6 observations pasted into data editor)

. metan pevent pnoevent uevent unoevent, label(namevar=authors, yearvar=publishedy) by(publicationtype) fixed or counts group1(PWB) group2(UWB)

Study | OR [95% Conf. Interval] % Weight

---------------------+---------------------------------------------------

RCT

Bodén (2004) | 1.000 0.054 18.574 2.85

Markmiller (2011) | 0.844 0.210 3.396 13.74

Unver (2004) | (Excluded)

Sub-total |

M-H pooled OR | 0.871 0.248 3.058 16.59

---------------------+---------------------------------------------------

non-RCT

Kishida (2001) | 1.442 0.319 6.529 8.90

Bernasek (2013) | 2.312 1.452 3.680 74.51

Rao (1998) | (Excluded)

Sub-total |

M-H pooled OR | 2.219 1.423 3.459 83.41

---------------------+---------------------------------------------------

Overall |

M-H pooled OR | 1.995 1.316 3.024 100.00

---------------------+---------------------------------------------------

Test(s) of heterogeneity:

Heterogeneity degrees of

statistic freedom P I-squared**

RCT 0.01 1 0.918 0.0%

non-RCT 0.34 1 0.559 0.0%

Overall 2.24 3 0.523 0.0%

** I-squared: the variation in OR attributable to heterogeneity)

Note: between group heterogeneity not calculated;

only valid with inverse variance method

Significance test(s) of OR=1

RCT z= 0.22 p = 0.830

non-RCT z= 3.52 p = 0.000

Overall z= 3.26 p = 0.001

-------------------------------------------------------------------------

**Stata commands for Table 3 and Figure 3.**

. *(11 variables, 7 observations pasted into data editor)

. metan pevent pnoevent uevent unoevent, label(namevar=authors, yearvar=publishedy) by(publicationtype) fixed or counts group1(PWB) group2(UWB)

Study | OR [95% Conf. Interval] % Weight

---------------------+---------------------------------------------------

RCT

Bodén (2004) | 6.176 0.260 146.777 0.79

Bottner (2005) | 1.545 0.087 27.358 1.50

Ström (2007) | 0.978 0.201 4.760 6.37

Markmiller (2011) | (Excluded)

Sub-total |

M-H pooled OR | 1.552 0.463 5.205 8.66

---------------------+---------------------------------------------------

non-RCT

Woolson (2002) | 5.426 0.247 118.958 0.93

Bernasek (2013) | 0.526 0.322 0.859 90.41

Kishida (2001) | (Excluded)

Sub-total |

M-H pooled OR | 0.576 0.358 0.927 91.34

---------------------+---------------------------------------------------

Overall |

M-H pooled OR | 0.661 0.425 1.026 100.00

---------------------+---------------------------------------------------

Test(s) of heterogeneity:

Heterogeneity degrees of

statistic freedom P I-squared**

RCT 1.06 2 0.589 0.0%

non-RCT 2.16 1 0.142 53.6%

Overall 5.10 4 0.277 21.5%

** I-squared: the variation in OR attributable to heterogeneity)

Note: between group heterogeneity not calculated;

only valid with inverse variance method

Significance test(s) of OR=1

RCT z= 0.71 p = 0.477

non-RCT z= 2.27 p = 0.023

Overall z= 1.85 p = 0.065

-------------------------------------------------------------------------

**Stata commands for Table 4 and Figure 4.**

. *(11 variables, 8 observations pasted into data editor)

. metan pevent pnoevent uevent unoevent, label(namevar=authors, yearvar=publishedy) by(publicationtype) fixed or counts group1(PWB) group2(UWB)

Study | OR [95% Conf. Interval] % Weight

---------------------+---------------------------------------------------

RCT

Markmiller (2011) | 3.405 0.339 34.168 14.25

Ström (2007) | 0.383 0.014 10.199 20.88

Monticone (2014) | 1.278 0.322 5.066 57.54

Shabana (2016) | (Excluded)

Sub-total |

M-H pooled OR | 1.403 0.485 4.057 92.67

---------------------+---------------------------------------------------

non-RCT

Chan (2001) | 5.364 0.246 116.761 7.33

Woolson (2002) | (Excluded)

Kishida (2001) | (Excluded)

Bernasek (2013) | (Excluded)

Sub-total |

M-H pooled OR | 5.364 0.246 116.761 7.33

---------------------+---------------------------------------------------

Overall |

M-H pooled OR | 1.693 0.634 4.525 100.00

---------------------+---------------------------------------------------

Test(s) of heterogeneity:

Heterogeneity degrees of

statistic freedom P I-squared**

RCT 1.19 2 0.552 0.0%

non-RCT 0.00 0 . .%

Overall 1.84 3 0.606 0.0%

** I-squared: the variation in OR attributable to heterogeneity)

Note: between group heterogeneity not calculated;

only valid with inverse variance method

Significance test(s) of OR=1

RCT z= 0.63 p = 0.532

non-RCT z= 1.07 p = 0.285

Overall z= 1.05 p = 0.294

-------------------------------------------------------------------------
